# Low Contrast Visual Acuity Might Help to Detect Previous Optic Neuritis

**DOI:** 10.3389/fneur.2020.602193

**Published:** 2020-12-22

**Authors:** Soo-Hyun Park, Choul Yong Park, Young Joo Shin, Kyoung Sook Jeong, Nam-Hee Kim

**Affiliations:** ^1^Department of Neurology, Department of Critical Care Medicine, Department of Internal Hospital, Inha University, Incheon, South Korea; ^2^Department of Ophthalmology, Dongguk University Ilsan Hospital and Dongguk University-Seoul Graduate School of Medicine, Goyang, South Korea; ^3^Department of Ophthalmology, Hallym University Medical Center, Seoul, South Korea; ^4^Department of Occupational and Environmental Medicine, Wonju Severance Hospital, Wonju, South Korea; ^5^Department of Neurology, Dongguk University Ilsan Hospital and Dongguk University-Seoul Graduate School of Medicine, Goyang, South Korea

**Keywords:** optic neuritis, visual acuity, low contrast visual acuity, neuromyelitis optica spectrum disorder, multiple sclerosis

## Abstract

Optic neuritis (ON) has been considered to be an important factor in the diagnosis of multiple sclerosis (MS) and neuromyelitis optica spectrum disorder (NMOSD), making ON detection increasingly critical for early diagnosis. Furthermore, subclinical ONs presenting no distinct decrease in visual acuity can be missed. Low contrast visual acuity (LC-VA) is known to be able to capture visual loss not seen in conventional high-contrast visual acuity (HC-VA) in MS. Therefore, to increase the sensitivity of ON detection, we investigated the advantage of LC-VA over conventional HC-VA. One hundred and eight patients with demyelinating disease (35 MS, 73 NMOSD) with ON at least 3 months prior and 35 controls underwent neuro-ophthalmic evaluation, including best-corrected conventional high contrast visual acuity (HC-VA) and 2.5% and 1.25% low contrast visual acuity (LC-VA). Receiver operating characteristic (ROC) curve analysis and the area under the curve (AUC) of various visual functions were used to determine the most relevant visual function test for the detection of optic nerve involvement. Additionally, the optimal cutoff point was obtained from the Youden index (J-index) as the points with the best sensitivity-specificity balance. When distinguishing ON from non-ON, the area under the ROC curve (AUC) was highest for the 2.5% LC-VA (0.835, *P* < 0.001; sensitivity 71.5%, specificity 88.6%), while it was 0.710 (*P* < 0.001) for the HC-VA and 0.770 (*P* < 0.001) for the 1.25% LC-VA. In discriminating between controls and ON, the AUC was also highest for the 2.5% LC-VA 0.754 (*P* < 0.001; sensitivity 71.5%, specificity 78.5%), while it was 0.719 (*P* < 0.001) for HC-VA and 0.688 (*P* < 0.001) for 1.25% LC-VA. In eyes with a history of ON (*n* = 137), the HC-VA and 2.5% LC-VA were abnormal in 64.2 and 71.5%, respectively (*P* < 0.001), with their combination detecting abnormalities in approximately 85.4% (*P* < 0.001). The 2.5% LC-VA was superior to HC-VA in detecting ON when distinguishing ON from non-ON or control. The 2.5% LC-VA might be a useful, feasible, and rapid method to detect ON. Furthermore, combining 2.5% LC-VA with conventional HC-VA would be better for detecting optic nerve involvements.

## Introduction

Optic neuritis (ON) has been reported to be accompanied by demyelinating diseases such as multiple sclerosis (MS) and neuromyelitis optica spectrum disorder (NMOSD). Although ON is often resolved after appropriate treatment (1), it can cause visual disturbance and reduce the quality of life (2). ON is related to retinal axon loss, and its morphological measurement is used as a parameter of disability (3). Demyelinating diseases sometimes may present subclinical changes in visual function, which impedes their early diagnosis (2, 4). The diagnosis of NMOSD and MS in the early phase is important for their treatments and prognoses. According to the recently revised criteria of MS and NMOSD (5, 6), ON has been regarded as a more important factor for their diagnoses (6). Additionally, asymptomatic or subclinical ONs are sometimes missed in the measurement of visual function with high contrast visual acuity (HC-VA), because of a normal result of HC-VA (3, 7). Therefore, it is challenging to diagnose MS or NMOSD with asymptomatic or subclinical ON. A lot of tests including optical coherence tomography (OCT), visual evoked potential (VEP), color vision, or visual field defect have been suggested to evaluate ON (8–12). Each test investigates a unique aspect of the visual system, and several variations of each test exist (8–12). The choice of the test depends on the purpose of the study, characteristics of the patient population, and types of diseases. Recent studies suggested that low-contrast visual acuity (LC-VA) could be a more sensitive measure of visual dysfunction in ON (8, 13, 14). LC-VA can be an easy, fast, and sensitive test to evaluate deficit in visual function caused by ON in a clinical setting. In our study, we aimed to investigate the usefulness of LC-VA as a diagnostic test for ON in a large cohort of demyelinating diseases.

## Materials and Methods

### Patients

This observational and cross-sectional study was performed according to the tenets of the Declaration of Helsinki and was approved by the Institutional Review Board of Dongguk University Ilsan Hospital. All subjects provided informed written consent. Patients with NMOSD who were seropositive for aquaporin-4 antibody, as defined by the revised 2006 diagnostic criteria of Wingerchuk, and patients with MS who met the 2010 McDonald criteria were recruited. Patients who had an episode of ON within the last 3 months were excluded to evaluate the utility of LC-VA for assessing evidence of remote ON in eyes with stabilized visual function after optic nerve inflammation. Patients with diabetes, a history of ocular injury, glaucoma, or other ophthalmologic disorders were excluded. Ophthalmological evaluations were performed in all patients by an ophthalmologist. Finally, 143 subjects were enrolled that consisted of 35 control participants and 108 patients (35 MS and 73 NMOSD).

### Visual Function

All visual tests were administered monocularly. Best-corrected conventional visual acuity (VA) with 100% contrast (high contrast visual acuity, HC-VA) was measured using the standard Snellen chart. Two-meter Sloan letter charts of 1.25 and 2.5% contrasts (Precision Vision, La Salle, IL) were used for LC-VA. LC-VA testing performed the discrimination of gradually smaller gray letters with 1.25 and 2.5% contrast level against a white background. Visual acuity (VA) was expressed using a decimal scale but was transformed to the logarithm of the minimum angle of resolution (logMAR) for statistical analyses.

### Statistical Analysis

Data are presented mean (standard deviation), min, max, median (interquartile range), number (percentage), and percentile (25th, 50th, and 75th). Comparisons between groups were performed using the Student *t*-test or Mann–Whitney test considering normality and the properties of the variables. ANOVA test was used to compare the means of three or more groups. Detection capacity of the diagnosis for ON was tested by the receiver operating characteristic (ROC) curve analysis, and area under the curve (AUC) was calculated to determine the discriminative value of each VA test. Sensitivity and specificity analyses were performed for the diagnosis of ON. In addition, the optimal cutoff point was obtained from the Youden index (J-index) as the points with the best sensitivity-specificity balance. VA was analyzed using the logarithm of the minimum angle of resolution (9). *P* < 0.05 were considered to be significant. All *P*-values reported are two-sided. SPSS 26.0 for Windows (SPSS Inc., Chicago, IL, USA) was used for the statistical analysis. R 4.0.1 for Windows (Washington University, St. Louis, MO, USA) were used for graph data.

## Results

**Figure 1:**
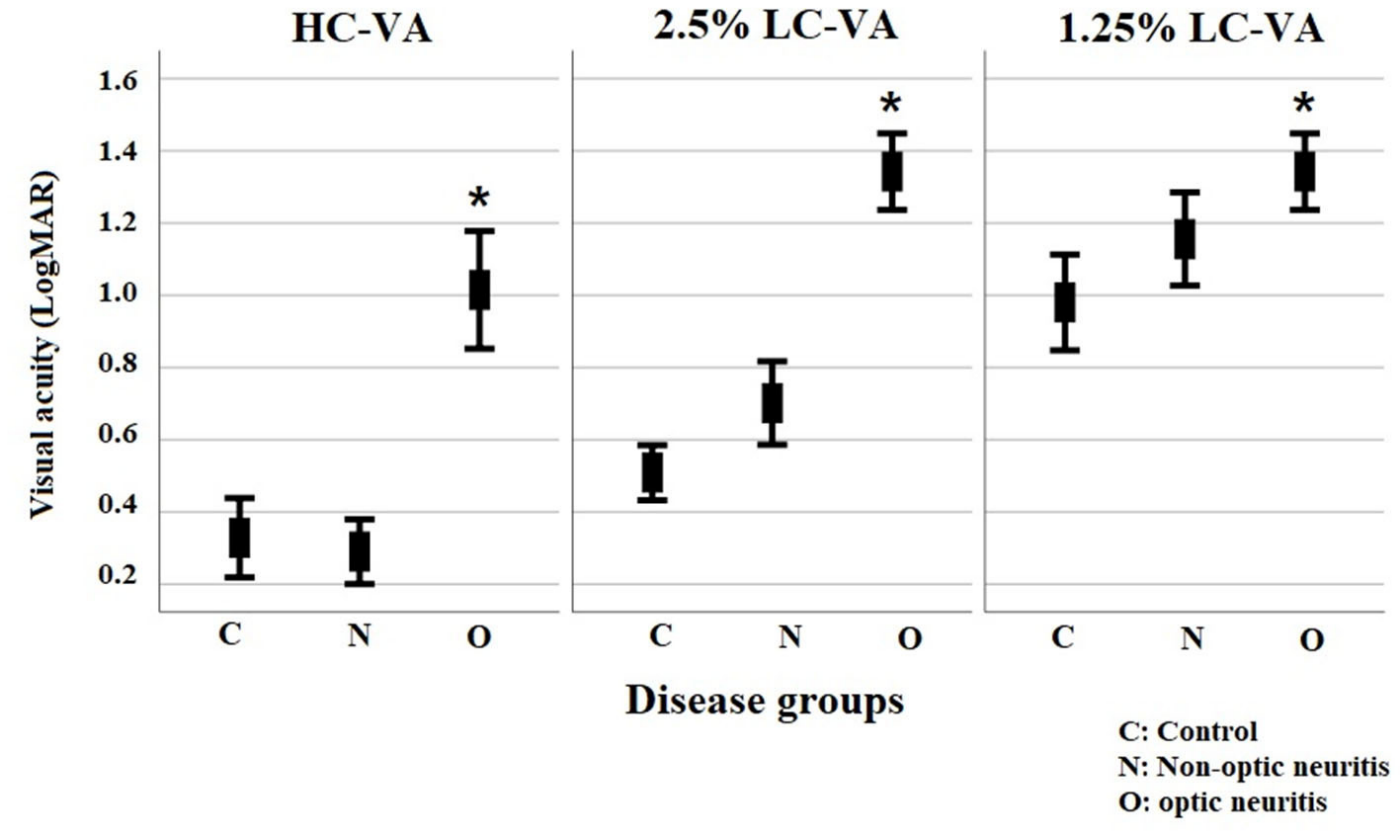
Visual functions for high-contrast visual acuity and for Sloan charts at 2.5% and 1.25% contrast levels. VA, visual acuity; HC-VA, high contrast visual acuity; LC-VA, low contrast visual acuity. *p < 0.05, control vs ON.

The clinical characteristics of each group are listed in Table 1. Total of 286 eyes (70 control eyes, 137 eyes with ON, and 79 eyes without ON) were assessed. Visual functions including HC-VA and 2.5 and 1.25% LC-VA in each group are shown in Table 2 and Figure 1. HC-VA was not significantly different between the control and non-ON groups, whereas HC-VA was worse in ON compared with the non-ON or control. Although 2.5% LC-VA and 1.25% LC-VA were not different between control and Non-ON, 2.5 and 1.25% LC-VA were worse in the ON group compared with the control or non-ON.

**Table 1:** Baseline characteristics according to study groups.

	**Control** **(*n* = 70)**	**Non-ON** **(*n* = 79)**	**ON** **(*n* = 137)**	***P*****-value**		
				**Control vs. non-ON**	**ON vs. non-ON**	**ON vs. control**
Age, mean ± SD (years)	39.3 ± 11.1	39.1 ± 11.7	37.7 ± 11.5	0.996	0.673	0.634
Female, *n* (%)	20 (28.6%)	61 (77.2%)	111 (81.0%)			
Diagnosis						
MS, *n* (%)		33 (41.8%)	37 (27.0%)			
NMOSD, *n* (%)		46 (58.2%)	100 (73.0%)			
Number of ON attacks, mean (95% CI)			2.09 (1.71–2.47)			
Bilateral ON, *n* (%)			116 (84.7%)			
Disease duration, mean ± SD (months)		53.2 ± 49.8	79.6 ± 52.2		<0.001	
EDSS, mean ± SD		2.7 ± 2.0	3.7 ± 2.0		<0.001	

*MS, multiple sclerosis; NMOSD, neuromyelitis optica spectrum disorder; ON, optic neuritis; Non-ON, non-optic neuritis; SD, standard deviation; EDSS, Expanded Disability Status Scale; CI, confidence interval*.

**Table 2:** Visual functions in LogMAR according to study groups.

	**Mean**	**Min**	**Max**	**Percentile**			***P*-value**
				**25th**	**50th**	**75th**	
**HC-VA**							
Control	0.3	−0.1	1.6	0.0	0.1	0.7	0.95^*^
Non-ON	0.3	−0.1	1.3	0.0	0.1	0.6	<0.001^**^
ON	1.0	−0.1	3.0	0.1	0.8	1.6	<0.001^***^
**2.5% LC-VA**							
Control	0.5	0.2	1.8	0.3	0.4	0.6	0.092^*^
Non-ON	0.7	0.2	1.8	0.3	0.6	0.7	<0.001^**^
ON	1.3	0.2	1.8	0.6	1.8	1.8	<0.001^***^
**1.25% LC-VA**							
Control	1.0	0.3	1.8	0.6	0.8	1.8	0.114^*^
Non-ON	1.2	0.3	1.8	0.7	0.8	1.8	<0.001^**^
ON	1.6	0.4	1.8	1.8	1.8	1.8	<0.001^***^

* Control vs. non-ON;

** Non-ON vs. ON;

*** *Control vs. ON. ANOVA in Scheffe*.

*HC-VA, high contrast visual acuity; LC-VA, low contrast visual acuity; ON, optic neuritis; Non-ON, non-optic neuritis*.

**Figure 2:**
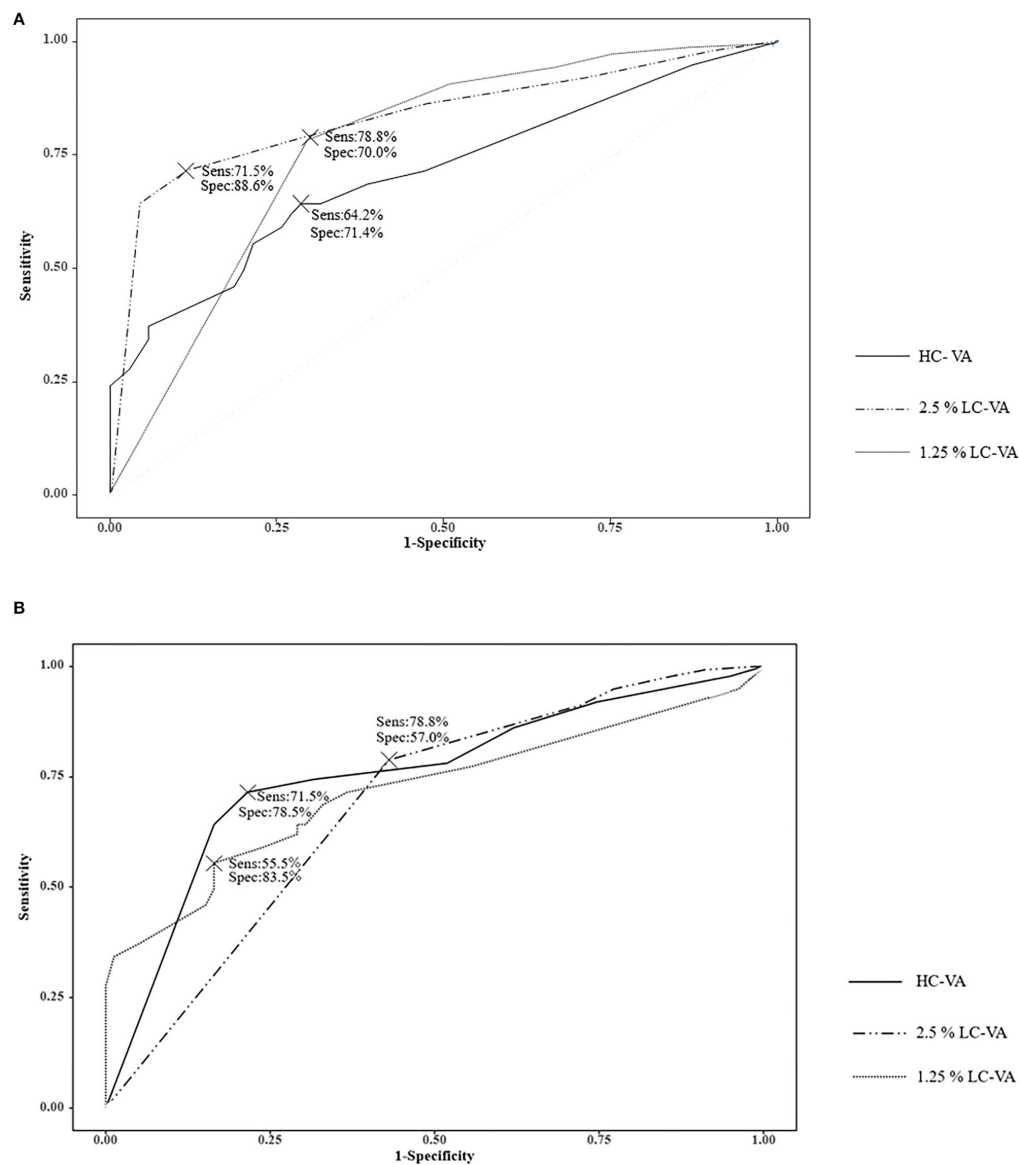
Receiver operating characteristic (ROC) curve. **(A)** ROC curve analysis of the visual functions to discriminate between ON and Non-ON. **(B)** ROC curve analysis of the visual functions to discriminate between controls and ON. Sens, sensitivity; Spec, specificity; VA, visual acuity; HC-VA, high contrast visual acuity; LC-VA, low contrast visual acuity.

The ROC curve analysis of the visual functions was used to determine the most appropriate test for discriminating between ON and non-ON (Table 3 and Figure 2A). The area under the ROC curve (AUC) was highest in the 2.5% LC-VA (sensitivity 71.5%, specificity 88.6%). AUC was 0.710 for the HC-VA, 0.835 for the 2.5% LC-VA, and 0.770 for the 1.25% LC-VA.

**Table 3:** Receiver operating characteristic curve analysis of visual functions to discriminate between ON and non-ON or control.

	**AUC (95% CI)**	***P*-value**	**Cutoff value**	**Specificity**	**Sensitivity**	**J-index**
**ON (*****n*** **=** **137) vs. non-ON (*****n*** **=** **79)**						
HC-VA	0.710 (0.654–0.775)	<0.001	0.450	71.4%	64.2%	0.357
2.5% LC-VA	0.835 (0.780–0.891)	<0.001	0.715	88.6%	71.5%	0.601
1.25% LC-VA	0.770 (0.697–0.843)	<0.001	1.300	70.0%	78.8%	0.488
**ON (*****n*** **=** **137) vs. control (*****n*** **=** **70)**						
HC-VA	0.719 (0.652–0.785)	<0.001	0.450	83.5%	55.5%	0.390
2.5% LC-VA	0.754 (0.687–0.821)	<0.001	0.750	78.5%	71.5%	0.500
1.25% LC-VA	0.688 (0.611–0.764)	<0.001	1.300	57.0%	78.8%	0.358

*HC-VA, high contrast visual acuity; LC-VA, low contrast visual acuity; ON, optic neuritis; Non-ON, non-optic neuritis; AUC, area under the receiver operating characteristic curve; CI, confidence interval*.

The ROC curve analysis of visual functions was used to determine the most appropriate test to discriminate between the controls and ON (Table 3 and Figure 2B). The AUC was also highest in the 2.5% LC-VA (sensitivity 71.5%, specificity 78.5%). The AUC was 0.719 for HC-VA, 0.754 for 2.5% LC-VA, and 0.688 for 1.25% LC-VA.

**Figure 3:**
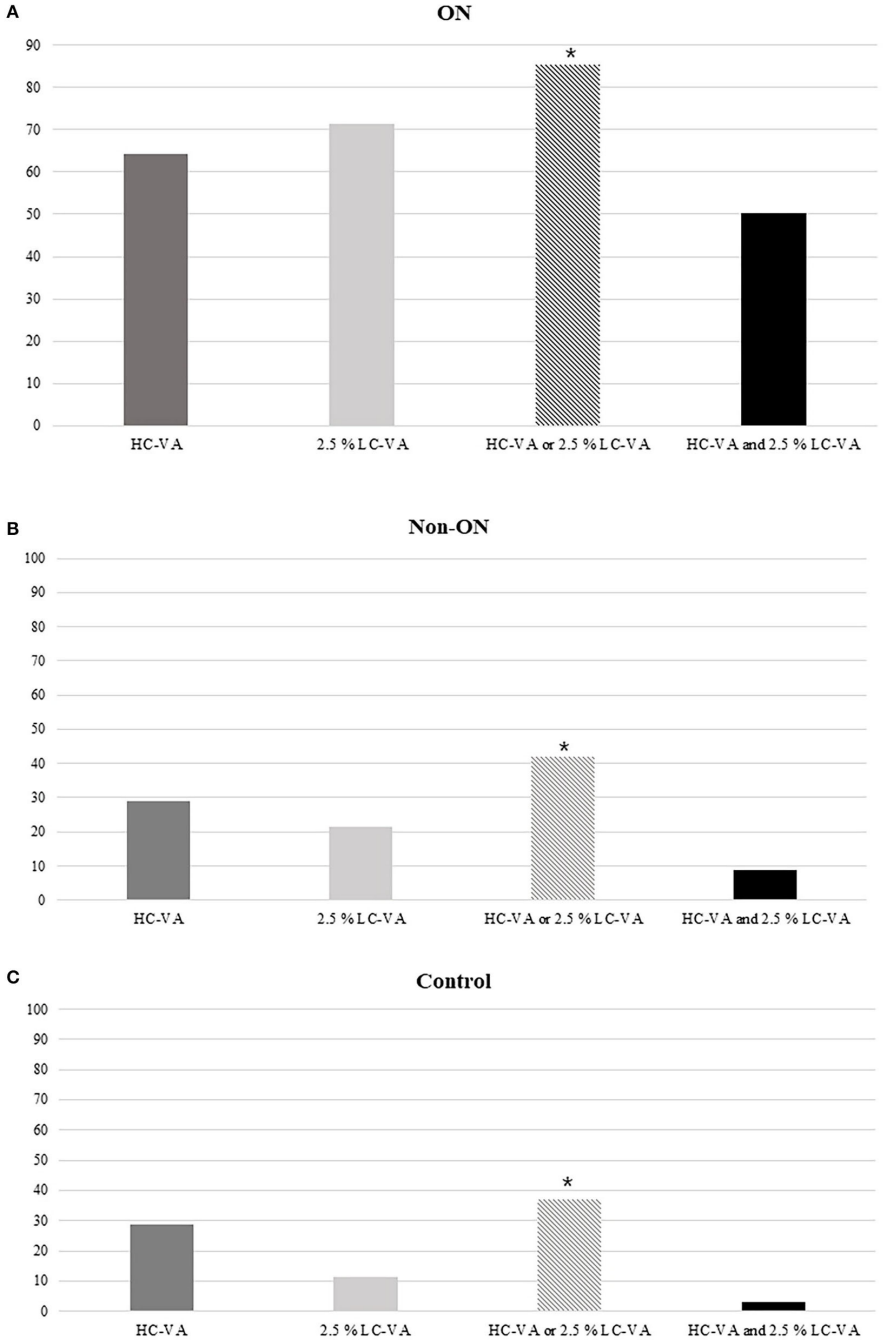
Percentages of abnormal tests. **(A)** Percentages of the abnormal tests in ON. **(B)** Percentages of abnormal tests in Non-ON. **(C)** Percentages of abnormal tests in the control. HC-VA or 2.5% LC-VA, eyes with at least one abnormal HC-VA or 2.5% LC-VA finding; HC-VA and 2.5% LC-VA, eyes with abnormal HC-VA and abnormal 2.5% LC-VA findings. ON, optic neuritis; non-ON, non-optic neuritis; VA, visual acuity; HC-VA, high contrast visual acuity; LC-VA, low contrast visual acuity. *p < 0.05, control vs ON.

The findings of HC-VA and 2.5% LC-VA were abnormal in 64.2 and 71.5% with a history of ON, respectively (Figure 3). Of the 137 eyes with ON, 19 (13.9%) were abnormal only in HC-VA, 29 (21.2%) were abnormal only in 2.5% LC-VA, and 69 (50.4%) were abnormal in both tests. The combination of HC-VA or 2.5% LC-VA detected abnormalities in 85.4% of ON and significantly improved the sensitivity relative to individual technique. Of the 79 eyes with non-ON, abnormalities were detected in 23 (29.1%) by HC-VA and 17 (21.5%) by 2.5% LC-VA. The combination of HC-VA or 2.5% LC-VA detected abnormalities in 33 (41.8%) of eyes and improved the sensitivity compared with those of each of the techniques individually.

## Discussion

This study showed that LC-VA was more sensitive compared with HC-VA for detecting ON. Our study supports the use of LC-VA in the detection of ON, given its high sensitivity and specificity, especially the use of 2.5% LC-VA. Moreover, the combination of HC-VA and 2.5% LC-VA was superior for the detection of ON than individually. This implies that 2.5% LC-VA may be a potential marker for ON.

Visual symptoms of ON may worsen as a result of various pathological processes, including inflammation, demyelination, and axonal degeneration of the visual pathway (15). Furthermore, discrimination of ON including subclinical ON is very important to assess the disease progression and recovery (16). Various diagnostic methods of ON have been studied for early detection of ON including VA, visual field, brain imaging, VEP, OCT, etc. (17). Although there are various tests for ON discrimination, the addition of the LC-VA test is easier and simpler to apply than the other tests (13, 18). Administering HC-VA and LC-VA tests requires a short testing time of approximately 5–10 min. Even though HC-VA has demonstrated normal results, patients with ON often complain of “discomfort” in their vision (13). HC-VA often did not differentiate slight changes in visual function by ON and cannot detect subtle visual disturbance or recovery over time (8, 19).

Previous studies investigated LC-VA and identified it to be a highly reliable visual assessment method in MS patients with and without ON (20). Additionally, the clinical relationship between ON or worsening LC-VA has been demonstrated (14, 19, 21), which suggests its role as an early indicator of ON associated with the visual disturbance (21). However, there have been limitations such as the lack of comparative studies in a large number of patients and being studied only in MS patients (19, 21). In our study, we applied the LC-VA test in a large number of patients with ON including more NMOSD patients than MS patients. We additionally analyzed the ROC in the subgroup of MS patients. As with the previous studies, 2.5% LC-VA was found to be most useful for detecting ON in the MS patient group (Supplementary Table 1). Thus, this study on a large number of demyelinating diseases added to evidence that LC-VA can be useful to detect significant visual dysfunction in both MS and NMOSD, especially in eyes with mild ON.

The very faint letters of 1.25% LC-VA are difficult to distinguish by healthy eyes. Our study also demonstrated that 2.5% LC-VA had high sensitivity and specificity for the detection of ON than did 1.25% LC-VA. Additionally, our study revealed that 2.5% LC-VA might detect subclinical ON that was missed by HC-VA in comparison with non-ON and control. Therefore, it is suggested that 2.5% LC-VA can be the most useful method to identify ON than HC-VA or 1.25% LC-VA.

A comparison of the sensitivity of these techniques (HC-VA and 2.5% LC-VA) has not been performed previously. As mentioned before, 1.25% LC-VA has a worse value of visual acuity even for the healthy eyes. Therefore, except 1.25% LC-VA, this study revealed a novel finding that the combination of HC-VA and 2.5% LC-VA significantly improved the sensitivity for the detection of ON than each of the techniques individually. These results are very important to make its use realizable in busy clinical settings or research for finding evidence to diagnose ON.

This study had several limitations. First, this was a single-center study. Additional multicenter studies should be conducted. Second, this study evaluated only Asians and more NMOSD patients than MS patients due to local epidemiological factors in South Korea. Therefore, in this study, a larger group of NMOSD could have a significant impact compared to the previous studies of other races. Third, our study was a retrospective study. Disease duration and number of events for individual patients varied significantly. We only analyzed the cases more than 3 months after ON. Therefore, our study helps to find remote ON attack evidence. Further study is needed for the utility of LC-VA in acute ON. Fourth, we could not calculate the cutoff value with the ROC curve of each visual test to discriminate between MS and NMOSD due to each group having eyes with very different visual severity. Retinal damage, including retinal nerve fiber layer and ganglion cell layer thinning, and VA is more severe with the number of ON attacks and relatively more severe in NMOSD than in MS (22). Therefore, very severe residual visual disturbance (<0.4 decimal in HC-VA) after the first episode of ON was suggested as an indicator of NMOSD compared with MS (23). If each group has large numbers of eyes with first ON presenting comparable visual severity, comparison of each visual test between MS and NMOSD can be possible. Further studies are warranted to investigate the cutoff values of LC-VA and its potential implications for the diagnosis in NMOSD or MS. Fifth, our study checked only the NMO-IgG in patients. Recently, myelin oligodendrocyte glycoprotein antibody (MOG-IgG) was found in a subset of NMOSD-IgG negative NMOSD patients, extending the range of NMOSD (24–26). Therefore, patients with MOG-IgG positive require further investigation.

In conclusion, our study suggests that LC-VA can better detect the visual disturbance in ON than HC-VA in NMOSD as well as MS (8, 11, 14, 19–21, 27). Considering all of those, 2.5% LC-VA might be the most useful, feasible, and rapid method to detect evidence of ON and could be used as a potential additive diagnostic tool of ON. HC-VA and 2.5% LC-VA test may yield more powerful result for ON detection in clinical practice.

## Data Availability Statement

The original contributions presented in the study are included in the article/Supplementary Materials, further inquiries can be directed to the corresponding author/s.

## Ethics Statement

The studies involving human participants were reviewed and approved by the Institutional Review Board of Dongguk University Ilsan Hospital. The patients/participants provided their written informed consent to participate in this study. Written informed consent was obtained from the individual(s) for the publication of any potentially identifiable images or data included in this article.

## Author Contributions

S-HP, CYP, YJS, KSJ, and N-HK: conception and organization of the research project. S-HP, CYP, YJS, and N-HK: execution of the research project. S-HP, KSJ, and N-HK: analysis and interpretation, and review and critique of the manuscript. S-HP and N-HK: writing of the first draft of the manuscript. All authors contributed to the article and approved the submitted version.

## Conflict of Interest

The authors declare that the research was conducted in the absence of any commercial or financial relationships that could be construed as a potential conflict of interest.
