# Supplementary material for: Low Contrast Visual Acuity Might Help to Detect Previous Optic Neuritis
Source: Front Neurol. 2020 Dec 22;11:602193. doi: 10.3389/fneur.2020.602193 (PMC7783398; doi:10.3389/fneur.2020.602193)
Supplement: Supplementary file 1 [file Table_1.DOCX]

**Supplementary Table 1. Receiver operating characteristic curve analysis of visual functions to discriminate between MSON and MSNON or control**

|  | **AUC**  **(95% CI)** | | **P value** | | **Cut-off value** | **Specificity** | | | **Sensitivity** | | **J-index** | | | |
| --- | --- | --- | --- | --- | --- | --- | --- | --- | --- | --- | --- | --- | --- | --- |
| **MSON (n= 33) vs. MSNON (n= 37)** | | | | | | | | | | | | | | |
| **HC-VA** | 0.496  (0.359-0.632) | 0.418 | | |  |  | | |  | |  | | | |
| **2.5% LC-VA** | 0.717  (0.595-0.839) | 0.005 | | | 0.750 | 87.9% | | | 59.5% | | 0.473 | | | |
| **1.25% LC-VA** | 0.690  (0.565-0.816) | < 0.001 | | | 0.650 | 39.4% | | | 91.9% | | 0.313 | | | |
| **MSON (n= 33) vs. Control (n= 70)** | | | | | | | | | | | | | | |
| **HC-VA** | 0.521  (0.404-0.638) | 0.372 | |  | | | |  |  | | | |  |  |
| **2.5% LC-VA** | 0.742  (0.631-0.852) | < 0.001 | | 0.750 | | | | 59.5% | 88.6% | | | | 0.480 |  |
| **1.25% LC-VA** | 0.702  (0.602-0.802) | < 0.001 | | 1.300 | | | 62.2% | | | 70.0% | | 0.322 | | |

HC-VA, high contrast visual acuity; LC-VA, low contrast visual acuity; MSON, multiple sclerosis with optic neuritis; MSNON, multiple sclerosis without optic neuritis; AUC, area under the receiver operating characteristic curve; CI, confidence interval
